# Salivary Cortisol Patterns in People Living With Dementia

**DOI:** 10.1093/geroni/igab046.1692

**Published:** 2021-12-17

**Authors:** Darina Petrovsky, Fanghong Dong, Liming Huang, Subhash Aryal, G Adriana Perez, Nancy Hodgson

**Affiliations:** 1 Rutgers University, Philadelphia, Pennsylvania, United States; 2 UPenn, PHILADELPHIA, Pennsylvania, United States; 3 University of Pennsylvania, Philadelphia, Pennsylvania, United States; 4 University of Pennsylvania School of Nursing, Philadelphia, Pennsylvania, United States; 5 University of Pennsylvania School of Nursing, Phildelphia, Pennsylvania, United States; 6 University of Pennsylvania, School of Nursing, philadelphia, Pennsylvania, United States

## Abstract

Salivary cortisol has a well-documented circadian pattern in older adults. Yet, the pattern of salivary cortisol in persons living with dementia (PLWD) due to circadian rhythm disturbances is unknown. This study examined diurnal salivary cortisol patterns in 176 PLWD (mean age 73.6±8.8, 33.3% male, clinical dementia rating >=0.5) by collecting saliva at waking (AM1), 30 minutes after waking (AM2) and bedtime (PM) over two consecutive days. Cortisol awakening response (CAR) was calculated as the change between AM2 and AM1 cortisol levels. The mean baseline salivary cortisol levels (ug/dl) were 0.35 (SD:0.3) at AM1, 0.40 (SD:0.39) at AM2, and 0.19 (SD:0.4) at PM. On average, cortisol levels decreased from morning to evening, with 58% exhibiting a positive CAR (mean 0.05; SD:0.34). There were no significant associations between cortisol levels with age, sex, obesity, and comorbidities. The findings demonstrated that diurnal cortisol rhythms are maintained in PLWD with a flattened CAR.

